# Predicting Moral Elevation Conveyed in Danmaku Comments Using EEGs

**DOI:** 10.34133/cbsystems.0028

**Published:** 2023-06-21

**Authors:** Chenhao Bao, Xin Hu, Dan Zhang, Zhao Lv, Jingjing Chen

**Affiliations:** ^1^ Department of Electronic Engineering, Tsinghua University, Beijing 100084, China.; ^2^ Department of Biomedical Engineering, Johns Hopkins University, Baltimore, MD, USA.; ^3^ Department of Psychiatry, School of Medicine, University of Pittsburgh, Pittsburgh, PA, USA.; ^4^ Department of Psychology, School of Social Sciences, Tsinghua University, Beijing 100084, China.; ^5^ School of Computer Science and Technology, Anhui University, Hefei 230601, China.

## Abstract

Moral elevation, the emotion that arises when individuals observe others’ moral behaviors, plays an important role in determining moral behaviors in real life. While recent research has demonstrated the potential to decode basic emotions with brain signals, there has been limited exploration of affective computing for moral elevation, an emotion related to social cognition. To address this gap, we recorded electroencephalography (EEG) signals from 23 participants while they viewed videos that were expected to elicit moral elevation. More than 30,000 danmaku comments were extracted as a crowdsourcing tagging method to label moral elevation continuously at a 1-s temporal resolution. Then, by employing power spectra features and the least absolute shrinkage and selection operator regularized regression analyses, we achieved a promising prediction performance for moral elevation (prediction *r* = 0.44 ± 0.11). Our findings indicate that it is possible to decode moral elevation using EEG signals. Moreover, the small-sample neural data can predict the continuous moral elevation experience conveyed in danmaku comments from a large population.

## Introduction

Affective computing is a field that employs machine learning methods to decode human emotions based on individuals’ behavioral or neurophysiological responses [1,2]. Recent researches have successfully decoded an individual’s emotions with neurophysiological signals recorded with electroencephalography (EEG) [3,4], functional magnetic resonance imaging (fMRI) [5,6], or functional near-infrared spectroscopy (fNIRS) [7,8]. While previous studies have proven the feasibility of affective computing based on brain signals, those studies have mainly focused on basic emotions (such as happiness/sadness/fear/surprise), with less exploration of emotions related to social cognition [9,10]. Considering the differences in theoretical construction, neurocognitive mechanisms, and practical applications between basic emotions and emotions associated with social cognition [11–15], it is important to investigate the feasibility of affective computing for social-related emotions.

The moral elevation is a social-related emotion elicited when witnessing others’ moral behaviors (e.g., doctors fighting against pandemics and saving a life) [16]. As a social emotion, moral elevation has been proven to play an important role in determining practical moral choices and behaviors in real life [17–19]. Previous studies have found that moral elevation could motivate prosocial behaviors such as inhibiting prejudice against gay men [20] and increasing donations to charity [21,22]. Moral elevation has also been suggested to improve well-being [23]. For example, when experiencing a higher moral elevation level, clinically depressed and anxious individuals reported a higher closeness to others, and lower interpersonal conflicts [24]. Recording daily moral elevation experiences on the Internet was also found to reduce depressive symptoms and increase happiness [25].

While moral elevation experiences are usually measured by self-reports, progress in the neural mechanism of moral elevation is expected to support an objective and automatic measurement for moral elevation. For example, Englander et al. [26] found that brain regions, including the medial prefrontal cortex (mPFC) and temporo-parietal junction (TPJ), were elicited specifically by moral elevation videos. Wang et al. [27] discovered co-activation of the left orbitofrontal cortex and left inferior temporal gyrus during picture-stimuli moral elevation experience. The specific neural patterns when experiencing moral elevation suggested the potential to decode moral elevation based on brain activities, yet the feasibility remains to be investigated.

The rapid-changing characteristic of affective experience also calls for attention when exploring the computing of moral elevation. Previous studies have often assumed that the affective state during a relatively long duration is stationary, such as tagging the whole video with the same affective label in video-based paradigms (usually for an epoch longer than 10 s) [2]. As the affective state can change rapidly at a scale of seconds [28,29], this stationary assumption may not always hold. Therefore, it is preferred to have continuous, dynamic affective labels to enable decoding with a higher temporal resolution rather than using the same affective label for a whole video. Some studies have tried to address this issue by a continuous self-report method. For example, participants needed to continuously move a sliding bar with a mouse for emotion labeling during the video [3,30]. However, these methods can be labor-intensive and time-consuming.

**Fig. 1.**
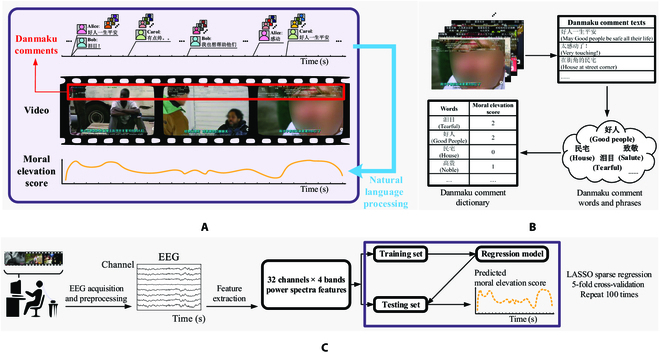
The experiment procedure and the pipeline of data processing. (A) The calculation for the temporal dynamics of moral elevation in the videos. Danmaku comments in each video for stimuli were first extracted as comment texts with time tags. Then, a natural language processing (NLP) method was performed to calculate the scores of moral elevation for each second of the videos using a danmaku comment dictionary. (B) The dictionary construction of danmaku comments. Danmaku comments from a cluster of moral elevation videos were extracted as plain texts without time tags. Texts were then segmented into single words or phrases. These words or phrases were rated in the pre-experiment to build a dictionary for moral elevation. (C) The pipeline for EEG processing. After pre-processing, EEG power spectra features for each second were calculated at theta, alpha, beta, and gamma. The LASSO regression with 5-fold cross-validation was performed to predict the dynamics of moral elevation conveyed in danmaku comments.

Internet-based crowdsourcing methods could offer a viable alternative for the continuous tagging of moral elevation [31–33]. One such method is danmaku comments, a popular type of commentary among Internet video audiences in East Asia [34]. Audiences can share their real-time comments anytime during their video watching, and their danmaku comments will be immediately displayed on top of the videos (see video screenshots in Fig. 1A), which are visible to other audiences. While each audience may only post danmaku comments at some discrete time points, continuous emotion tagging for the whole video could be achieved by accumulating audience numbers (for example, when more than 10,000 views). Moreover, the audience’s emotional experience is one of the most frequently conveyed information in the danmaku comment [34]. Previous studies have proved the feasibility of extracting public-level emotions based on danmaku comments. For example, Li et al. [35] proposed a framework that could identify multi-dimensional emotions from danmaku comments using natural language processing; He et al. [36] revealed the potential of danmaku comments in promoting public crisis communication during COVID-19. These studies suggest the possibility of danmaku comments to tag moral elevation continuously.

The present study aimed to explore the feasibility of affective computing for moral elevation based on brain signals. A continuous moral elevation tagging at a 1-s time resolution was achieved based on danmaku comments. EEG was used as the neuroimaging technique due to its high temporal resolution [37]. Twenty-three participants were invited to watch videos that were expected to elicit moral elevation with their EEG signals recorded. The least absolute shrinkage and selection operator (LASSO) regularized regression was employed to predict the temporal dynamics of moral elevation using EEG power spectra features. Our results demonstrated the feasibility of decoding the moral elevation conveyed in danmaku comments using EEGs.

## Methods

### Acquisition of danmaku comments and construction of danmaku comment dictionary

Following the practice in the setup of emotion stimuli datasets [38,39], 8 research assistants were invited to empirically select videos that could elicit moral elevation from bilibili.com (one of the most popular online danmaku video media platforms in China, with about 80,000,000 daily active users in average) according to the criteria of over 90,000 views and over 60 danmaku comments per second. Ten videos with real-time danmaku comments were selected after the primary screening. The number of videos is comparable with previous studies [38,39].

All danmaku comments of the 10 videos before 29 July 2020 were crawled using a customized Python script with an HTTP request module. These comments were segmented into single words or phrases using Jieba, a Chinese word segmentation tool [40]. Words or phrases without emotional information, such as negators, stop words, and degree words, were excluded according to the dictionary construction principle of previous NLP studies [35,41], leaving 321 words and phrases for the dictionary. Nevertheless, the negators and degree words were also considered during emotion tagging; see more details in the “Moral elevation tagging with danmaku comments” section.

Then, 138 participants (76 females, mean age = 22 years, ranging from 17 to 33 years old) were invited to rate all the 321 extracted words and phrases using 7-point Likert scales on the dimensions of the touched feeling and elevation. The moral elevation scores of those words and phrases were calculated as the mean rating scores of touched feeling and elevation averaged among the participants [42]. The moral elevation scores were further empirically re-coded into 0, 1, and 2, corresponding to the calculated scores of <2.5, 2.5 − 5.5, and >5.5. A moral elevation dictionary was thus established, with 321 words and phrases that were scored 0, 1, or 2 according to the moral elevation ratings, as illustrated in Fig. 1B.

### Selection of video stimuli in EEG recordings

After constructing the danmaku comment dictionary, these 10 videos obtained after the primary screening were edited to preserve the most moral elevating scenes. The duration of videos were 79, 136, 133, 108, 60, 149, 107, 84, 80, and 104 s, respectively. The 10 videos were then presented to a group of 49 participants (30 females, mean age = 23 years, ranging from 18 to 32 years, non-overlapping with the above experiments’ groups) to further validate whether these videos could elicit moral elevation. As a part of a larger project, participants were asked to report their emotional experiences after watching each video, with 7-point Likert scales on the dimensions of joy, sadness, disgust, anger, surprise, fear, touched feeling, elevation, valence, and arousal. Following the method in Ref. [42], 3 videos with the highest average ratings of touched feeling and elevation (6.60 ± 0.19, 6.55 ± 0.17, and 6.41 ± 0.26 respectively) were selected out of 10 videos as stimuli for moral elevation in the following EEG experiment. The 3 videos’ contents were about the charity behaviors of a disabled beggar, assistance in a medical emergency from strangers, and the fighting against a flood to protect people. The duration of the selected videos were 136, 84, and 80 s, summing to 300 s in total.

### Participants for EEG recordings

We recruited 23 college students from Tsinghua University with normal hearing and normal or corrected-to-normal vision for our study (10 females, mean age = 21 years, ranging from 17 to 24 years). All participants signed the informed consent forms voluntarily and received financial remuneration. The complete study, including preliminary and EEG experiments, was conducted following the Declaration of Helsinki and approved by the local Ethics Committee of the Department of Psychology, Tsinghua University (Protocol No. 201906).

### Experiment procedure

As a part of a larger project, these 23 participants were invited to watch 24 emotion-stimuli videos, including 3 moral elevation videos and 3 neutral videos (i.e., documentaries about tool manufacturing or everyday scenery in cities). The emotional videos were presented randomly in order. Participants were required to keep their heads and bodies steady during the video watching. After watching each video, participants reported their emotional experiences on 10 dimensions ranging from 0 to 7, including joy, sadness, disgust, anger, surprise, fear, elevation, touched feeling, arousal, and valence. The inter-video interval was 30 s.

Then, participants’ self-reported continuous emotional ratings during video watching were also obtained following the practice of a previous study [3]. Specifically, after watching all the videos, participants were asked to rate their continuous real-time emotional experiences to 5 randomly chosen replayed videos for a second time. This time, a vertical sliding bar was presented alongside the video screen, and the participants could freely and continuously drag the bar with the computer mouse during video watching to rate their real-time feelings of the to-be-evoked emotion, with the larger *y*-axis coordinate of the vertical sliding bar marked as the stronger emotion. Each moral elevation video was rated by 5 to 6 participants.

### Moral elevation tagging with danmaku comments

The moral elevation dictionary was then applied to the danmaku comments from the 3 selected videos for stimuli to tag the moral elevation experience. The moral elevation experience was calculated within each 1-s non-overlapping time window as the weighted sum of the moral elevation scores of all the words and phrases within this period. Each word or phrase was then graded by the dictionary score (i.e., 0, 1, or 2, as explained above), multiplied by a different weight. The weighted sums were further normalized within each video by dividing the total number of danmaku comments in this video to reach the final score. The calculation can be described as Eq. 1:

$$\text{moral elevation scor}e_{i}=\frac{\sum_{j=1}^{N}‍w_{j}\times \text{danmaku phrase scor}e_{j}}{N}$$ (1)

where *i* indicates the *i*th second of this video and *j* indicates the *j*th danmaku word or phrase in the *i*th second. *w_j_* indicates the weight of the *j*th danmaku word or phrase. The weight was empirically coded into −1 if an associated negator such as “not” was identified, and 0.75, 1.25, 1.5, or 2 for different levels of degree words such as “a little”, “more”, “very”, and “most”. *N* indicates the total number of danmaku comments in this video. In this way, the public’s moral elevation experience was obtained for each 1 s of all 3 selected videos.

Equation 1 was designed to integrate important danmaku comment features and reflect them on the moral elevation scores. While the dictionary scores of each word and phrase in the danmaku comments explicitly appeared in Eq. 1, the number of danmaku comments also makes contributions to the moral elevation score calculation from the following 2 aspects: firstly, the total number of the danmaku comments posted throughout the whole video is indicated as *N* and used as a data normalization to make the scores more robust to different videos with different amounts of danmaku comments; secondly, the number of danmaku comments within the 1-s segment implicitly contributes to the moral elevation scores by accumulating the scores from more words and phrases, as the danmaku comment temporal concentration becomes denser.

After calculating the moral elevation scores for each 1-s segment within each video using Eq. 1, we further applied data normalization on the inter-video level by computing the *z*-scores over all of the 1-s segments among the 3 videos to compare them on a unified scale. The pipeline of moral elevation tagging is illustrated in Fig. 1A and B.

### EEG recording and analysis

During the procedure, EEG signals were recorded using a 32-channel wireless EEG system (NeuSen. W32, Neuracle, China). The international 10-20 system was utilized to guide electrode placement (Fp1/2, Fz/3/4/7/8, FC1/2/5/6, Cz/3/4, CP1/2/5/6, T3/4/5/6, Pz/3/4, PO3/4, Oz/1/2, and A1/2 [left/right mastoids]). The reference electrode was placed at CPz, and the forehead ground was placed at AFz. Electrode impedances were maintained below 10 kOhm throughout the entire experiment. The sampling rate was set to 250 Hz.

The recorded EEG data were first notch filtered to remove the 50-Hz powerline noise, then bandpass filtered to 0.05 to 47 Hz. Independent components analysis was applied to remove artifacts related to eye movement. About 1 to 2 independent components were excluded from each participant’s EEG. Data were then filtered into the frequency bands of theta (4 to 7 Hz), alpha (8 to 13 Hz), beta (14 to 29 Hz), and gamma (30 to 47 Hz). The filtered signals were segmented into non-overlapping 1-s segments corresponding to the moral elevation experiences tag. The sum of the squares of 250-point values in each 1-s segment was then calculated to obtain the power spectra per channel per frequency band as the feature for the follow-up analysis, leading to 32 (channel) × 4 (frequency band) = 128 feature dimensions per second.

As the emotion tagging was extracted from public-based danmaku comments, these EEG features were also averaged across participants to obtain the group-level EEG features. The approach was suggested to effectively reflect the group-level emotional experience [3,38]. Finally, 128-dimensional EEG features were obtained for each second of each video.

Due to the relatively high-dimensional data feature (i.e., 128 dimensions) and the relatively small sample size (i.e., 300 s), the LASSO regression was employed for feature selection and regression model building. The LASSO regression model is shown as Eq. 2:

$$\begin{array}{ll} \hat{y} & =w^{T}x+b\\ w & =\text{argmin}{\left|y-w^{T}x-b\right|}_{2}+\lambda {\left|w\right|}_{1} \end{array}$$ (2)

where ***y*** indicates a 1 × 300 (s) vector of the moral elevation scores, ***w*** indicates a 128 × 1 vector of LASSO coefficients, ***x*** indicates a 128 × 300 features matrix, ***b*** indicates a shared bias, and *λ* is a hyperparameter for penalty.

Then, a 5-fold cross-validation was conducted to evaluate the prediction performance of the EEG-based moral elevation decoding. Specifically, the 300 128-dimension EEG features derived from the EEG responses when watching the moral elevation videos were split into 5 folds randomly, with 4 folds as the training sets and 1 fold as the testing set. Pearson’s correlation between the danmaku-based and LASSO-predicted moral elevation scores and the prediction Normalized Mean Squared Error (NMSE) were calculated. Then, the lambda that minimized NMSE in the training set was chosen to apply to the testing set for prediction. The procedure was repeated 5 times to get 5 cross-validated *r* values. To avoid the potential bias and imbalance brought by the data split, we further repeated the whole LASSO regression procedures 100 times. The averaged *r* value was considered as the prediction performance.

## Results

**Fig. 2.**
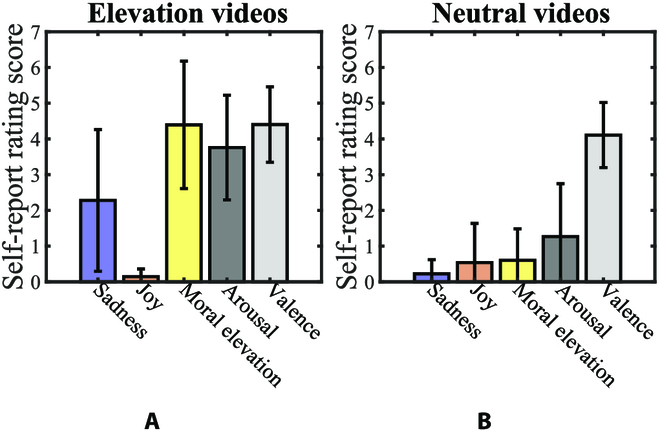
(A) The self-report ratings of sadness, joy, moral elevation, arousal, and valence for moral elevation videos. (B) The self-report ratings of sadness, joy, moral elevation, arousal, and valence for neutral emotion videos. The error bars indicate standard errors.

The self-report ratings for the moral elevation videos and the neutral videos are shown in Fig. 2. The “sadness” and “joy” dimensions were displayed because these 2 emotions were mostly mentioned and compared in previous moral elevation studies [11,16]. A paired *t*-test with Bonferroni correction was conducted to analyze the scores for each index between the neutral and moral elevation videos. Compared to the neural videos, the moral elevation videos elicited higher moral elevation (*P* < 0.001), indicating the effectiveness of the stimuli. In addition, the moral elevation videos were more aroused than the neutral videos (*P* < 0.001), but the 2 types of videos did not differ in their valence dimension (*P* = 0.148). Besides, the sadness dimension was scored higher in the moral elevation videos compared with the neutral video (*P* < 0.001), while the joy dimension was scored lower (*P* = 0.009).

**Fig. 3.**
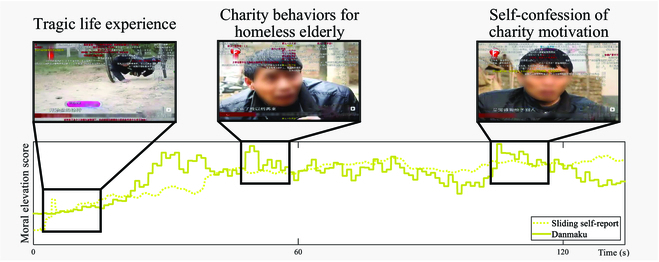
The temporal dynamics of moral elevation scores in a representative video (>300,000 views, >25,000 danmaku comments). The solid line represents the moral elevation scores calculated from danmaku comments. The dotted line represents the scores from real-time tagging averaged across 5 participants’ sliding reports. Pearson correlation *r* value reached 0.67 between scores from 2 different tagging methods, *P* < 0.001. Three screenshots in the video were demonstrated in chronological order, respectively: the tragic life experience of the protagonist; his charity behaviors for the homeless senior citizens; his self-confession of charity motivation.

Figure 3 demonstrates the temporal dynamics of moral elevation scores calculated from danmaku comments and self-reports in a representative video (>300,000 views, >25,000 danmaku comments). Similar trends between the scores of danmaku comments and self-reports could be observed (Pearson correlation *r* = 0.67, *P* < 0.001), suggesting the effectiveness of danmaku-based moral elevation scores. Three time periods are highlighted with screenshots in the video: the first period introduces the tragic life experience of the protagonist; the second period records his charity behaviors for the homeless senior citizens; the third period records his self-confession of charity motivation.

**Fig. 4.**
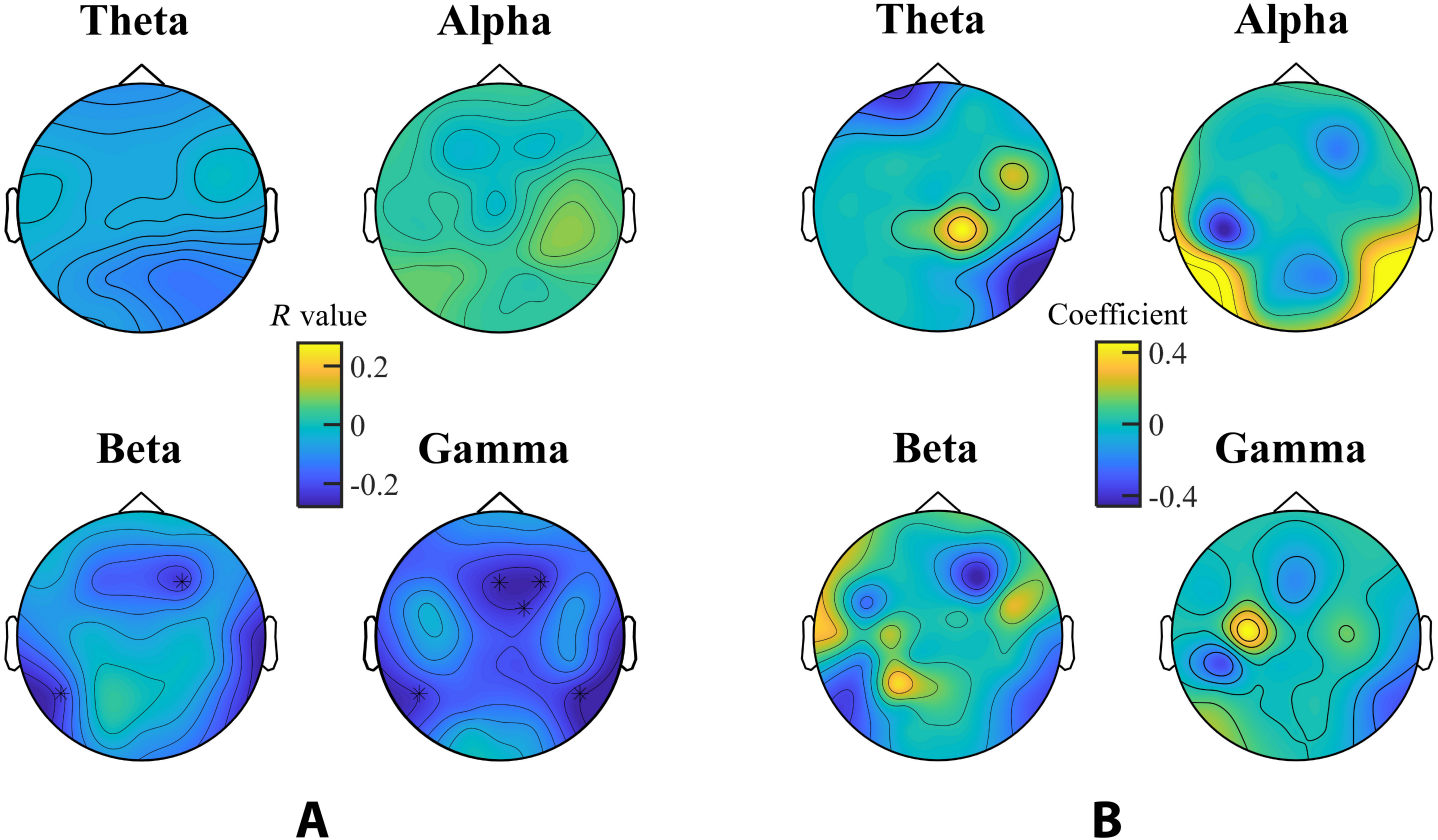
(A) The correlations between EEG power spectra features and the temporal dynamic of moral elevation scores. The correlation analysis was conducted in the theta, alpha, beta, and gamma bands separately. The stars indicated significance (*P* < 0.05) with Bonferroni correction. (B) The topographies show the selected features from the LASSO regression model of moral elevation.

Then, the correlation between the temporal courses of the group-level EEG power spectra features and the danmaku-based moral elevation scores is shown in Fig. 4A. Significant negative correlations were found in the power spectra of beta and gamma bands at frontal and bilateral temporo-parietal electrodes (beta band: *r* =  −0.23, *P* < 0.001 at F4 and *r* =  −0.22, *P* < 0.001 at P7; gamma band: *r* = −0.27, *P* < 0.001 at Fz, *r* =  −0.26, *P* < 0.001 at F4, *r* =  −0.24, *P* < 0.001 at FC2, *r* =  −0.24, *P* < 0.001 at P7, and *r* =  −0.28, *P* < 0.001 at P8; Bonferroni correction). No significant correlations were found at the theta band or alpha band.

**Fig. 5.**
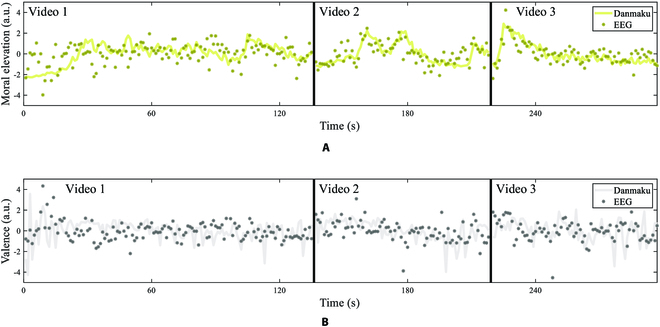
The regression results for the prediction of (A) moral elevation and (B) valence scores in the 3 moral elevation videos. The semitransparent solid lines are the danmaku-based scores calculated from the danmaku comments using danmaku dictionaries. The scattered dots are the EEG-based scores calculated from the group-level EEG features using the LASSO regression model.

Then, the LASSO regression achieved the cross-validated Pearson correlational *r* value at 0.44 ± 0.11 (mean ± standard deviation, calculated from 100 repetitions of 5-fold cross-validation) between the danmaku-based and LASSO-predicted moral elevation scores, corresponding to a prediction NMSE of 0.81 ± 0.17. The selected features for the moral elevation LASSO regression model re-emphasized beta and gamma bands over the frontal and bilateral temporo-parietal electrodes and highlighted theta band over the left prefrontal and right parietal electrodes, together with the alpha band over the bilateral parieto-occipital electrodes, shown in Fig. 4B. The predicted moral elevation scores were shown in Fig. 5A.

The LASSO regression was also applied to the prediction of valence with EEG power spectra features as a comparison. The cross-validated Pearson correlational *r* value was 0.45 ± 0.11, with a prediction NMSE of 0.87 ± 0.39. The predicted valence scores are shown in Fig. 5B. The LASSO regression for moral elevation achieved comparable predictive performance for valence.

## Discussion

Recent studies in affective computing have primarily focused on decoding basic emotions using brain signals. However, the decoding of moral elevation, a social emotion elicited when witnessing others’ moral behaviors, has yet to be explored. The present study achieved an EEG-based decoding of moral elevation for the first time, while combining a danmaku-based continuous tagging method to capture the rapid-changing nature of affective experience. To ensure interpretability in the decoding process rather than pursuing a high decoding accuracy, we opted for a straightforward LASSO-based method to demonstrate the feasibility of decoding moral elevation using EEG data. Indeed, our results suggested that it was possible to achieve the EEG-based decoding of moral elevation through such a simple method. More importantly, the high interpretability of LASSO offers the opportunity to take a closer look at EEG features that contribute to the decoding: we found that spatial patterns of the EEG features selected by LASSO could echo previous neuroscience studies that demonstrated the moral-elevation-related brain areas, thus providing the neural basis supporting the decoding. Our results suggested that it is possible to decode moral elevation at a 1-s temporal resolution based on EEG signals. While comparing different decoding models is beyond the scope of the present study, future studies that integrate the advanced models are expected to further boost the decoding of moral elevation.

In the present study, EEG correlates for the temporal dynamic of moral elevation were reported for the first time. Significant correlations of moral elevation were found in frontal and temporo-parietal electrodes, which were also highlighted in the features selected in the LASSO regression analysis. Englander et al. [26] discovered that mPFC and TPJ are activated by moral elevation videos in the fMRI recordings. As the aftereffects of moral elevation experience included reduced self-awareness and increased prosocial motivation [11,16,42], the engagements of mPFC and TPJ could be explained by the functional role of mPFC in dispositions of others and self, or interpersonal norms and scripts, and TPJ in temporary states such as goals and intentions [43]. Our study added supportive evidence about the involvement of frontal and temporo-parietal areas when experiencing moral elevation from the modality of EEG. Moreover, by decomposing the EEG signal into different frequencies, significant correlations with moral elevation were seen at beta and gamma bands, which might be explained by their functional roles in general affective processing reported in previous studies [44–46]. Besides, although no significance with moral elevation scores was observed in either theta or alpha band with univariable correlation analysis, the multi-variable LASSO analysis highlighted the contributions of the alpha band at bilateral parietal electrodes and theta band at the left prefrontal electrodes as well as the right parietal electrodes. In previous EEG-based hyperscanning studies, the alpha-band activities at right centro-parietal regions showed inter-brain synchrony when people interacted during joint actions [47]. The contribution of alpha band features might be explained by its role in social interaction. At the same time, a decrease of theta oscillations over the right parieto-occipital clusters was found to correlate with greater sharing intention in previous studies [48], while theta activation covering the left orbitofrontal cortex was reported during morally bad judgment conditions [49]. These might together explain the contribution of theta band features. While exploring the physiological mechanism behind moral elevation experience has been continuing, our study provided EEG correlates of moral elevation from a computing perspective.

Furthermore, although moral elevation can be considered a positive emotion due to its potential benefits for well-being [23], it is important to note its differences from the classic positive emotion of happiness. Previous studies usually used comedic or funny videos to elicit positive emotions [3,50]; the videos used in the present study, however, elicit moral elevation by contrasting suffering with moral behaviors (such as the charitable actions of a disabled beggar). Thus, moral elevation here should not be explained as the classic “joy positive”, but the “inspiration positive” [38], as supported by participants’ relatively high ratings in the “sadness” dimension and low ratings in the “joy” dimension. Further research exploring the distinctions between different types of positive emotions is needed to provide insight into the concept of “positivity” [38,51].

The present study also demonstrated the feasibility of danmaku comments as a source for crowdsourcing tagging, which offers several advantages over traditional self-report tagging methods. First, danmaku comments were posted by audiences spontaneously during their daily video-watching activities, which could provide a higher ecological validity than self-reports collected during experimental settings. Second, with the development of NLP techniques, affective states could be calculated automatically from the video’s danmaku comments, thereby saving time and labor for continuous self-reports. Third, danmaku-based tagging usually involved a large number of crowds on the Internet. As our findings demonstrated that EEG data from 23 participants could effectively predict the moral elevation conveyed in danmaku comments, it suggested the possibility of predicting moral behaviors in a large population with small-sample neural data [52–54].

Establishing quantitative methods to measure psychological states has long been a challenging problem in computational psychology, known as the inverse problem [55]. As the first study to investigate the inverse problem of decoding moral elevation based on EEG recording, there is room for further improvements. First of all, the classical power spectra features were used in the present study. Decoding performance is expected to benefit from more complex features and advanced machine learning methods in further studies. Moreover, while the present study aimed to decode moral elevation based on crowd-level tagging, more studies on the intra-individual level were expected in the future.

Electroencephalography-based decoding of moral elevation, defined as the emotion elicited when witnessing others’ moral behaviors, was investigated in a video-watching paradigm among 23 participants. Our study shed light on danmaku comments as a crowdsourcing tagging source for continuous labeling moral elevation. Regression analyses revealed a promising decoding performance, suggesting the potential of using small-sample neural data to predict moral elevation experience conveyed in danmaku comments from a large population.

## Data Availability

Data are available upon reasonable request.
